# Brain abscess and epidural empyema caused by *Salmonella enteritidis* in a child: successful treatment with ciprofloxacin: a case report

**DOI:** 10.1186/1757-1626-2-7131

**Published:** 2009-06-05

**Authors:** Daniel Blázquez, Miriam Muñoz, Celia Gil, Jose Luis Ruibal, Firdaus El Knaichi, Esther Aleo

**Affiliations:** Department of Pediatrics, Hospital ClínicoSan Carlos, 28040, MadridSpain

## Abstract

Focal intracranial infections caused by *Salmonella* are rare, especially those produced by *S. enteritidis*. We describe the case of a 26-month-old girl who underwent surgery for a frontoparietal ependymoma and presented with epidural empyema and a brain abscess due to *S. enteritidis* following an episode of gastroenteritis. The child was successfully treated by surgical drainage along with 9 weeks of antibiotic therapy including ciprofloxacin.

## Introduction

Despite bacteremia, sepsis and meningitis being relatively common in infants, episodes of focal intracranial infection produced by Salmonella are rare. Torrey et al. [1] reported an incidence of up to 6% bacteremia in infants under 12 months old with salmonellosis and Rocha described a 1.3% rate of meningitis in newborns under 18 months with this disease [2].

The literature contains only 80 described cases of focal intracranial infection by Salmonella, including cerebral abscesses and subdural and epidural empyemas. In these infections, Salmonella typhi is the most frequently isolated causative agent [3]. Brain abscesses caused by Salmonella enteritidis are especially rare, and only 12 episodes have been described in the literature, 3 of these affecting children [4,5]. The present case report is the first description of a Salmonella infection producing both epidural empyema and brain abscess.

## Case presentation

**Figure 1.**
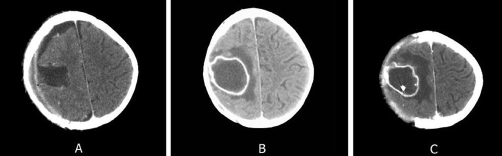
**(A)** Right parietal epidural empyema caused by Salmonella enteritidis. **(B)** Right frontoparietal brain abscess caused by Salmonella enteritidis. **(C)** Drainage tube placed in the abscess.

A 26-month-old girl underwent surgery for a grade II right frontoparietal ependymoma, in which the tumor was completely resected. Dexamethasone (1 mg/kg/day) was given 8 days after surgery as antiedema treatment. Seven days after the operation, the child suffered an episode of vomiting and diarrhea that lasted 24 hours. Two days later, she presented at the Emergency unit with a fever of 39°C. Her general state was good, with no neurological signs or other accompanying symptoms. Of the blood tests performed on hospital admission, the following results are of interest: 12000 leucocytes per mm^3^, with 72% neutrophils and 8 mg/dl and C-reactive protein (CRP) (normal < 0.5 mg/dl). On suspicion of a possible complication of surgery, a cranial CT-scan was requested, which showed a fluid collection indicative of a right epidural parietal empyema (Figure 1A). Through a right parietal craniectomy, the epidural fluid collection was drained and a sample obtained for culture. After placement of a subdural drainage tube the patient was started on empirical antibiotic treatment with ceftazidime and vancomycin.

Cultures of the epidural empyema fluid, cerebrospinal fluid and feces revealed the growth of Salmonella enterica, subspecies enterica I, serotype Enteritidis 9, 12: g, m, phage type 6a.

In all the cultures, MICs for ceftazidime (1 ≤ μg/mL), cefotaxime (1 ≤ μg/mL), ciprofloxacin (1 ≤ μg/mL), nalidixic acid (4 ≤ μg/mL), and chloramphenicol (8 ≤ μg/mL) indicated a good sensitivity of the bacterium to these antibiotics.

On the sixth day of empirical antibiotic treatment, the patient continued to have fever and clonic jerking started in the left arm. A further CT scan revealed a right frontoparietal lesion with rim enhancement consistent with a cerebral abscess (Figure 1B).

A drainage tube was placed in the abscess (Figure 1C), and anticonvulsant therapy with valproic acid initiated. At this time point, vancomycin was discontinued and ceftazidime was replaced with i.v. chloramphenicol and this new antibiotic regimen continued for 4 weeks. In the fluid evacuated from the abscess, an identical strain of *Salmonella enteritidis* as in the previous cultures was identified. Twenty four hours after the introduction of chloramphenicol and placement of the new drainage tube, the infant's temperature returned to normal and she suffered no more seizures. Given that *Salmonella enteritidis* continued to appear in cultures of the abscess drainage fluid, on day 38 of treatment, chloramphenicol was replaced with i.v. ciprofloxacin and i.v. cefotaxime. This regimen was maintained for 2 weeks. The patient was discharged after 7 weeks of intravenous antibiotic treatment and was given oral ciprofloxacin at home for a further 15 days.

Lesion progression was monitored weekly by transcraniectomy cerebral ultrasonography and monthly by cerebral NMR. These follow up exams revealed a gradual reduction in abscess size until its resolution 9 weeks after the onset of antibiotic therapy.

At 10 months, the previously extracted and sterilized bone fragment was replaced by cranioplasty. After two years of follow up, the child is well with no signs of infection or tumor recurrence observed in the CT or NMR scans. She has no neurological or development alterations and her weight and height are within the 50th percentile for her age.

## Discussion

The genus Salmonella has two species, *Salmonella enterica* and *Salmonella bongori*, although only the first of these causes diseases in humans. *Salmonella enterica* includes more than 2300 serovars, which are classified as six genogroups (subspecies) I-VI. Most strains pathogenic for man (causing gastroenteritis and typhus) are serotypes of subspecies I.

Intracranial focal lesions due to Salmonella can present at any age, though subdural and epidural empyemas are more common in children than adults. In general, their prognosis is poor with a mortality rate as high as 40% described in some patient series [6].

There is often a series of predisposing factors for developing an intracranial lesion due to Salmonella including: associated meningitis due to Salmonella, primary or metastatic brain tumors, subarachnoid hemorrhage, epidural or subdural hematoma, neurosurgery and immunodeficiency [7]. The patient described here had been recently operated on for a low grade supratentorial ependymoma, and it was a residual epidural collection of fluid following neurosurgery that triggered the infection. The same microbe was isolated from the stools and in cultures of the empyema and cerebral abscess. It is likely that the bloodstream was the route of spread from the enteric focal infection. Immunological determinants in our patient were normal.

The treatment of choice for brain abscesses caused by Salmonella is long-duration antibiotic therapy along with surgical drainage [8]. Salmonella is an intracellular pathogen, so antibiotics acting as this level are required. Traditionally, chloramphenicol has been used to treat brain abscesses caused by this bacterium, since the drug is capable of crossing the blood-brain barrier and adequately disseminates through the CNS [6]. However, a fear of possible side-effects and increasing microbial resistance have largely limited its use in favor of an increased use of third generation cephalosporins. In our patient, intravenous treatment with ceftazidime followed by chloramphenicol along with surgical drainage managed to considerably reduce the size of the abscess, but despite the good sensitivity of the agents in vitro, this treatment combination was unable to completely eradicate the bacterium, which continued to appear in the abscess drainage fluid after 4 weeks of treatment. In contrast, the inclusion of ciprofloxacin in the antibiotic regimen was able to completely resolve the child's symptoms. Wessalowski [9] described the case of a neonate with multiple brain abscesses caused by *Salmonella enteritidis*, who required the use of intravenous ciprofloxacin after treatment with cefotaxime and chloramphenicol had failed despite sensitivity shown towards the two drugs in vitro. As in our case, the patient also progressed favorably in response to ciprofloxacin.

Recent studies have failed to find evidence of permanent cartilage lesions in children receiving fluoroquinolones, and the number of indications of fluoroquinolones has increased in pediatric practice to the extent that they are currently an accepted treatment option for severe Salmonella infection [10,11].

In the case described here, early surgical drainage together with intravenous long-duration antibiotic therapy including ciprofloxacin served to completely resolve the intracerebral lesions present.

## List of abbreviations

CNS: Central Nervous System
CRP: C-reactive protein
CT: Computer tomography
i.v: intravenous
MIC: Minimum Inhibitory Concentration
NMR: Nuclear Magnetic Resonance
